# Insuring Babies' Lives

**Published:** 1914-04-18

**Authors:** Sidney Webb


*To the Editor of* The Hospital.

### Insuring Babies' Lives.

*To the Editor of* The Hospital.

Sir,—Will you allow me to appeal for information and suggestions in an inquiry of some public interest? The Fabian Research Department, in continuing its study of insurance, is now investigating the working of industrial life assurance (the widespread system of canvassing households to insure their children's lives), and the provision of funeral benefit by friendly societies, co-operative societies, clubs, and trade unions, together with the system of compensation for accidents now so generally made a matter for insurance by employers. We want to know the good effects and the bad of the canvassing system, which makes industrial insurance so costly.

We want to know whether the insured persons always get what is due to them, and if not, on what grounds is payment refused? We want to know the good effects and the bad of insuring babies' lives; and whether there is now any injurious "gambling in lives." We want to know about the lapsed policies, why they lapse, who profits by their lapse and who loses, and what surrender value or fully-paid-up policy is given to the policyholder in exchange for the lapsed policy.

We want to know why millions of poor people prefer the expensive policies of the companies to the cheaper policies of the Post Office.

We want to know how the co-operative insurance works, under which every store member finds himself and his wife automatically insured, in proportion to their purchases, without having to pay any weekly pennies at all.

I should be grateful of any account of personal experience, or any description of how these things are working, and for any suggestions for their improvement. A full list of points for inquiry will be sent on application.—I am, Sir, yours faithfully,

Sidney Webb.

[We deal with Mr. Webb's request in a Note on page 59 of this issue.—Ed., The Hospital.]

